# A patient‐derived xenograft and a cell line derived from it form a useful preclinical model for small bowel adenocarcinoma

**DOI:** 10.1002/cam4.2986

**Published:** 2020-03-13

**Authors:** Tomoki Yamano, Shuji Kubo, Naohiro Tomita

**Affiliations:** ^1^ Division of Lower Gastrointestinal Surgery Department of Surgery Hyogo College of Medicine Nishinomiya Japan; ^2^ Laboratory of Molecular and Genetic Therapeutics Institute for Advanced Medical Sciences Hyogo College of Medicine Nishinomiya Japan

**Keywords:** oxaliplatin, patient‐derived xenograft, preclinical model, rare tumor, small bowel adenocarcinoma

## Abstract

Basic and clinical studies on small bowel adenocarcinoma (SBA) are limited due to the rare nature of this cancer. We established a patient‐derived xenograft (PDX) model from the tumor tissue of an advanced SBA patient with liver and peritoneal metastasis, and a cell line from the PDX. In the PDX model, compared to the control group, 5‐fluorouracil (5‐FU) treatment resulted in statistically significant tumor growth inhibition (TGI), while oxaliplatin (OHP) and irinotecan had no significant inhibitory effects. In combination with 5‐FU, OHP showed the highest rate of TGI. The IC_50_ for OHP was significantly lower than those for paclitaxel, gemcitabine, and trifluorothymidine in the PDX‐derived cell line when compared to in HT29, a colon cancer cell line. Genetic analysis of the patient tumor, PDX tumor, and the cell line demonstrated consistency in the microsatellite status and mutations in *TP53*, *APC*, *HRAS*, *CSF1R*, *FGFR3*, *FLT3*, *PDGFRA*, and *RET* genes. However, the PDX tumor alone had additional mutations, indicating that the PDX‐derived cell line may support the unstable genetic status of the PDX. Our findings confirmed the effectiveness of the combination of OHP and 5‐FU, which is a common treatment for advanced SBA and advanced colorectal cancer, in a preclinical model. This preclinical model of SBA can help in further understanding the biology of SBA.

## Graphical abstract

Our data indicated that 5‐FU was the key drug for treatment and OHP alone had the additional effect in combination with 5‐FU.

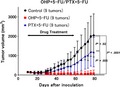

## 1 INTRODUCTION

Small bowel malignancies are rare tumors, with an estimated 10,470 new cases diagnosed annually, and approximately 1,450 deaths in the United States.^1^ Small bowel adenocarcinoma (SBA) is one of the most common small bowel malignancies.^2^, ^3^ Most cases are detected at an advanced stage because SBA is rare and difficult to diagnose.^4^, ^5^ Retrospective studies have shown that combination therapy with oxaliplatin (OHP) and 5‐fluorouracil (5‐FU) is the most effective treatment for advanced SBA.^6^, ^7^ Prospective phase II studies have also suggested the usefulness of this combination.^8^, ^9^, ^10^ However, SBA being rare, there have been limited phase III clinical trials and basic research to confirm the usefulness of this combination therapy. Even new technologies such as next‐generation sequencing have not helped in confirming the benefits of this combination therapy. Therefore, preclinical models of rare tumors such as SBA are required to understand their biology better.

Patient‐derived xenograft (PDX) was proposed as a preclinical model for experimental chemotherapy more than 30 years ago.^11^ Recently, PDXs have been re‐evaluated as powerful preclinical models for combined drug sensitivity and genetic analysis.^12^ Most PDXs are derived from common malignancies such as colorectal, pancreatic, breast, or lung cancer.^13^, ^14^ However, PDXs derived from rare tumors such as malignant pleural mesothelioma and gastrointestinal stromal tumor have been reported, which may be good preclinical models for these tumors .^15^, ^16^

In this study, we established and evaluated a PDX as a preclinical model for SBA. We also established a cell line from the PDX. This preclinical model for SBA could help develop novel treatments for this rare tumor.

## 2 MATERIALS AND METHODS

### 2.1 Patient background

A 74‐year‐old woman was referred to ABC College of Medicine Hospital with a weight loss of 10 kg. She was diagnosed with advanced SBA of the jejunum with liver, peritoneal, and lymph node metastasis. She underwent surgery for resection of the primary tumor, followed by systemic chemotherapy. The initial chemotherapy included nine cycles of FOLFOX (folinic acid + 5‐FU + OHP). After a diagnosis of progressive disease using computed tomography, 11 cycles of FOLFIRI (folinic acid + 5‐FU + irinotecan) were administered, although this treatment was also ineffective. One year after the surgery, she died of SBA. This study was conducted in accordance with the guidelines of the Ethics Committee of ABC College of Medicine, and the experimental protocols involving the patient's sample and genetic analysis were approved by the Institutional Review Board of ABC College of Medicine (#0295). Written informed consent was obtained from the patient to use her tissue samples for research.

### 2.2 Patient‐derived xenograft

All animal experiments were performed in accordance with the guidelines for Animal Experiments of the Institutional Animal Care and Use Committee and were approved by the Institutional Committee for Ethics of Animal Experiments (#15‐064). Small pieces of tissue (5‐7 mm in diameter) were removed from the surgical specimens derived from both the primary and peritoneal tumors (Passage 0; P0) and stored in a cell bank (Nippon Zenyaku Kogyo) at −80°C until engraftment. Four‐week‐old female Balb/c nu/nu mice were purchased from Japan SLC (Hamamatsu). Mouse husbandry and experiments were performed under pathogen‐free conditions at the Institute of Experimental Animal Sciences of ABC College of Medicine. Mice were anesthetized using a combination of medetomidine hydrochloride (Nippon Zenyaku Kogyo Co., Ltd.), midazolam (Sandoz KK), and butorphanol tartrate (Meiji Seika Pharma Co., Ltd.). Small pieces of tissue were transplanted subcutaneously into the flanks of the mice (P1). We verified and measured the tumors twice weekly for at least 3 months after the initial transplantation. A PDX was considered to be established when the tumors implanted into the P2 mice grew successfully to ≥10 mm in diameter.^12^

### 2.3 Assessment of drug sensitivity using the PDX model

The small pieces of the tumor were subcutaneously transplanted into the right and left flanks of the mice. Each treatment group had 4‐5 mice. Tumor volume was calculated twice weekly as follows: a × b^2^, where “a” is the tumor length, and “b” the width.^12^ When the tumor diameter was >5 mm, the mice were randomized into no treatment (control) or drug treatment groups after adjusting the mean tumor volume among the groups. If the tumor formation was insufficient, the tumors were excluded from the randomization. The rate of tumor growth inhibition (TGI) was calculated as follows: 1 – (increase in tumor volume in the drug treatment group)/ (increase in tumor volume in the control group). TGI was assessed one week after the completion of drug treatment. The tumor volumes were then compared between the control and treatment groups using a t‐test. Effects of monotherapy were assessed by administering 10 mg/kg of OHP (Nipro), 20 mg/kg of irinotecan (Towa Pharmaceutical), or 50 mg/kg of 5‐FU (Kyowa Hakko Kirin) intraperitoneally once a week, for a total of eight weeks, into P3 mice, as previously described.^17^, ^18^, ^19^ The dose of irinotecan was determined to be twice that of OHP, based on the doses used clinically in humans. For assessment of combination therapy, 10 mg/kg of OHP, 20 mg/kg of irinotecan, or 12.5 mg/kg of paclitaxel (PTX, Nihonkayaku) was administered intraperitoneally with 50 mg/kg of 5‐FU once a week, for a total of eight weeks into P3 and P5 mice for OHP + 5‐FU, P3 mice for irinotecan + 5‐FU, and P5 mice for PTX + 5‐FU. PTX was included in the study based on a report by Overman et al^20^ showing the effects of nab‐paclitaxel in refractory SBA patients and in a PDX model from an SBA patient.

### 2.4 Culture of PDX‐derived cells and drug sensitivity in vitro

The tumor from P3 mice was mechanically minced and enzymatically lysed using a tumor dissociation kit and a tumor dissociator (Miltenyi Biotec Japan). SBA cells were collected using mouse depletion antibody and column separation (Miltenyi Biotec Japan). The human colon cancer cell line HT29 was a kind gift from Dr Hirofumi Yamamoto (Osaka University Graduate School of Medicine, Japan). HT29 was authenticated by ATCC using DNA profiling. Cells were maintained in RPMI supplemented with 10% fetal bovine serum, 10,000 units of penicillin, 10 mg/mL of streptomycin, and 25 μg/mL of amphotericin B. L‐glutamine was added for SBA cells to a final concentration of 2 mmol/L. Culture media and L‐glutamine were purchased from Nacalai Tesque (Kyoto, Japan). Antibiotic‐antimycotic solution and fetal bovine serum were obtained from Life Technologies Japan (Tokyo, Japan). All cells were grown at 37°C in a humidified incubator with 5% CO_2_. The SBA and HT29 cells were seeded in 200 μL of medium in 96‐well flat‐bottom plates at a density of 8 × 10^3^ cells per well. The next day, the medium was removed, and various concentrations of OHP (0.05 − 50 μmol/L), irinotecan (0.1 − 100 μmol/L), 5‐FU (0.1 − 100 μmol/L), and PTX (0.0001 − 1 μmol/L) were added. After drug treatment for 96 hours, cells were counted using a cell counting kit (Dojindo Laboratories, Kumamoto, Japan) in accordance with the manufacturer's instructions. The half‐maximal inhibitory concentration (IC_50_) was calculated as the concentration that corresponded to a 50% reduction in cellular growth compared with the untreated cells. Experiments were performed at least three times independently, and the data are presented as mean ± standard deviation. We also found that subcutaneous inoculation of 2 × 10^6^ SBA cells into mice resulted in successful tumor formation (in eight of eight mice).

### 2.5 Evaluation of PDX as a preclinical model

The effects of chemotherapy in the patient vs. the PDX models were compared. The clinical effects of chemotherapy were evaluated using the Response Evaluation Criteria in Solid Tumors (RECIST)^21^ as well as the expression of tumor markers carcinoembryonic antigen and cancer antigen 19‐9. Pathological assessment of the PDXs was performed by hematoxylin–eosin staining.

### 2.6 Genomic assessment of the PDX model

Genomic DNA from the primary tumor, PDX (P4), and PDX‐derived cell line was used to evaluate microsatellite instability and the mutation status of cancer‐associated genes. Oncogenes and tumor suppressor genes were analyzed by Ion AmpliSeq Cancer Hotspot Panel v2 to evaluate the mutation status during the process of generating the PDX from the patient tumor and cell line from the PDX (Lifetechnologies Japan, Tokyo, Japan). The mononucleotide microsatellite markers BAT‐25, BAT‐26, NR21, NR22, and NR24, were used for evaluation, as previously described.^22^

### 2.7 Statistical analysis

Statistical analyses were performed using JMP version 11 (SAS Japan Inc, Tokyo, Japan). Following drug treatment, we compared the tumor volumes in the PDX models of SBA and IC_50_ values in HT29 cells (in culture), using the Student's t‐test and Wilcoxon rank sum test. *P*<.05 were considered statistically significant.

## 3 RESULTS

### 3.1 Establishment of a PDX model for SBA

PDXs (Passage 1; P1) grew successfully from the primary and peritoneal tumors of an SBA patient. The P1 tumors were implanted into P2 mice. The PDX from the primary tumor was successfully implanted in up to P5 mice, while the metastatic peritoneal tumors were implanted in up to P4 mice at 16 months after implantation from the patient.

### 3.2 Drug sensitivity in the PDX model

**FIGURE 1:**
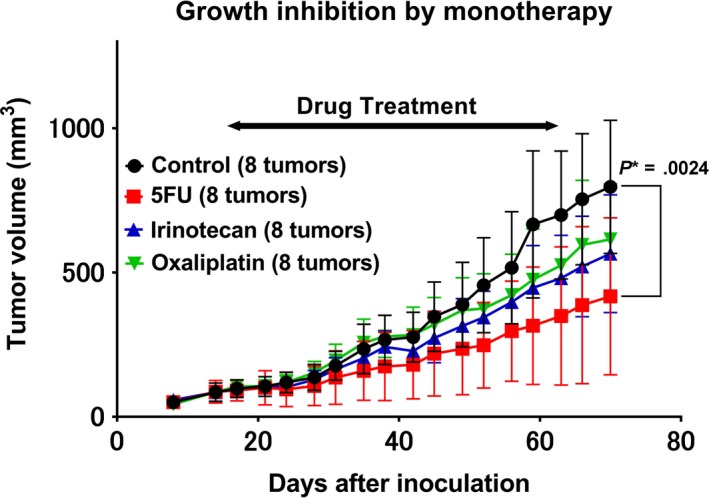
Monotherapy‐induced growth inhibition in the patient‐derived xenograft. In response to monotherapy with oxaliplatin, irinotecan, and 5‐fluorouracil, the tumor growth inhibition rate is 0.23, 0.31, and 0.52, respectively. Eight tumors from four mice were assessed for each treatment. 5‐FU treatment shows statistically significant growth inhibition compared to the control group (*P* = .0024). P* indicated *P* < .05

**FIGURE 2:**
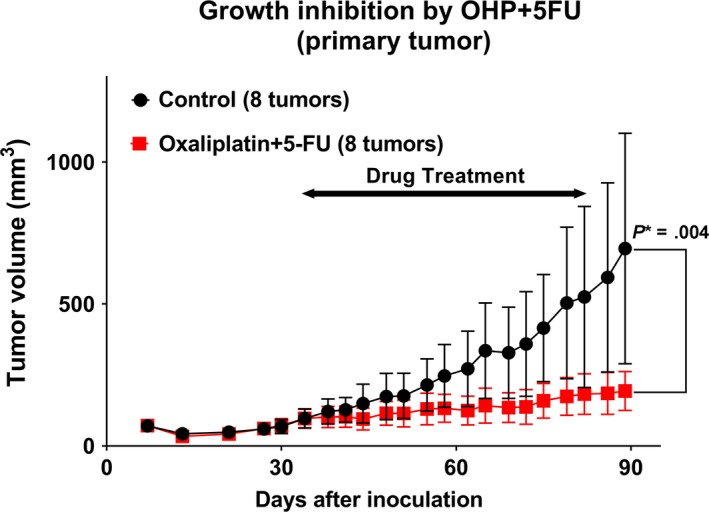
Combination therapy‐induced growth inhibition in the patient‐derived xenograft. Combination therapy with oxaliplatin and 5‐fluorouracil (5‐FU), results in tumor growth inhibition rate of 0.84 in the PDXs. Eight tumors from five mice were assessed. Oxaliplatin +5‐FU treatment shows statistically significant growth inhibition compared to the control group (*P* = .004). P* indicated *P* < .05

**FIGURE 3:**
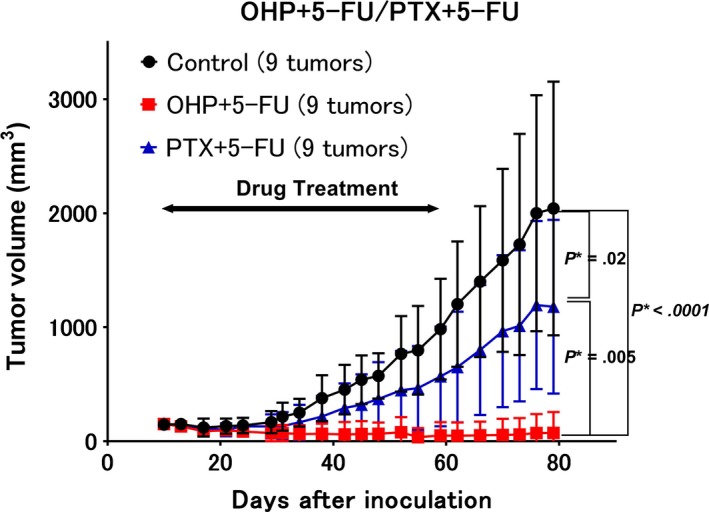
Comparison of different combination therapies. Shown is the growth inhibition following combination therapies with oxaliplatin +5‐fluorouracil (5‐FU) vs. paclitaxel +5‐FU. The growth inhibition rates following treatments with paclitaxel +5‐FU and oxaliplatin +5‐FU are 0.49 and 1.1, respectively. Nine tumors from five mice were assessed. The combination of oxaliplatin +5‐FU shows statistically significant growth inhibition compared to the control (*P* < .0001) and paclitaxel +5‐FU (*P* = .005) groups. The combination of paclitaxel +5‐FU shows statistically significant growth inhibition compared to the control group (*P* = .02). P* indicated *P* < .05

**FIGURE 4:**
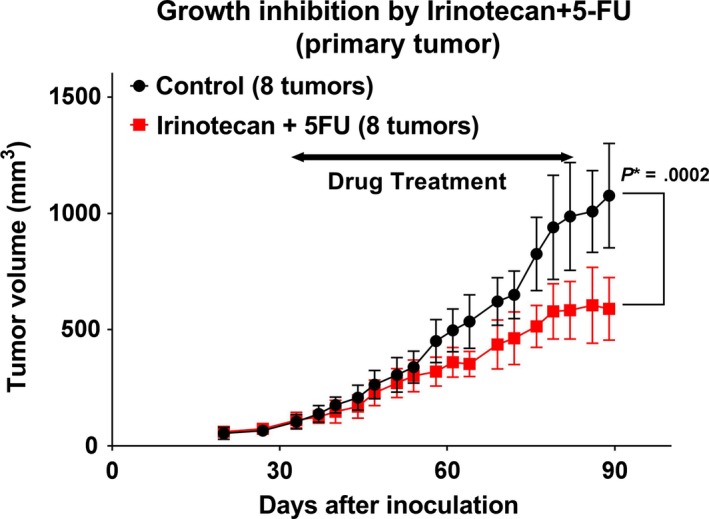
Combination therapy with irinotecan and 5‐fluorouracil in the patient‐derived xenograft. The tumor growth inhibition rate following treatment with irinotecan +5‐fluorouracil is 0.46. Eight tumors from six mice in the control group and eight tumors from five mice in the combination group were assessed. The combination treatment shows statistically significant growth inhibition compared to the control group (*P* = .00002). P* indicated *P* < .05

When tested individually, OHP, irinotecan, and 5‐FU showed a TGI rate of 0.23, 0.31, and 0.52, respectively (Figure 1). 5‐FU treatment resulted in statistically significant growth inhibition compared to the control group (*P* = .0024). When compared to 5‐FU alone (0.52 in Figure 1), OHP in combination with 5‐FU showed an increase in the rate of TGI (0.84 in Figure 2 and 1.1 in Figure 3), while PTX (0.49 in Figure 3) and irinotecan (0.46 in Figure 4) in combination with 5‐FU showed no increase in the rate of TGI. OHP + 5‐FU treatment resulted in statistically significant growth inhibition compared to PTX + 5‐FU (*P* = .005). All combinations showed significant growth inhibition compared to the control group (OHP + 5‐FU: *P* = .004 in Figure 2 and *P* < .0001 in Figure 3, PTX + 5‐FU: *P* = .02 in Figure 3, irinotecan + 5‐FU: *P* = .00002 in Figure 4). Our data, therefore, indicate that 5‐FU is the key drug for the treatment of SBA, and OHP has an additive effect in combination with 5‐FU.

### 3.3 Drug sensitivity in vitro

The sensitivity of the SBA cells generated from the PDX to different drugs was assessed in vitro. Table 1 shows the IC_50_ values for OHP, irinotecan, 5‐FU, PTX, gemcitabine (GEM), and trifluorothymidine (TFT) (Table 1). The IC_50_ of PTX, GEM, and TFT was better in HT29 cells than in the SBA cells, while that of OHP was better in the SBA cells than in HT29 cells. The IC_50_ values of irinotecan and 5‐FU were comparable in both the SBA and HT29 cells.

**TABLE 1:** The sensitivity of SBA and HT29 cells to different drugs

Drug	Cell	IC_50_ (μmol/L) mean ± SD	*P* Value
**Oxaliplatin**	SBA	0.40 ± 0.08	**.034**
	HT29	0.99 ± 0.16	
Irinotecan	SBA	9.86 ± 2.01	.3
	HT29	6.60 ± 2.85	
5‐FU	SBA	2.53 ± 2.7	.61
	HT29	0.91 ± 0.37	
**Paclitaxel**	SBA	0.055 ± 0.034	**.02**
	HT29	0.0036 ± 0.0014	
**Gemcitabine**	SBA	0.059 ± 0.013	**.0495**
	HT29	0.0047 ± 0.0003	
**Trifluorothymidine**	SBA	65.1 ± 43.6	**.0495**
	HT29	2.1 ± 0.54	

Note

The selected drugs with *P* < .05 are shown in bold.

Abbreviations: 5‐FU, 5‐fluorouracil; SBA, small bowel adenocarcinoma; SD, standard deviation

### 3.4 Evaluation of the PDXs as a preclinical model for SBA

**FIGURE 5:**
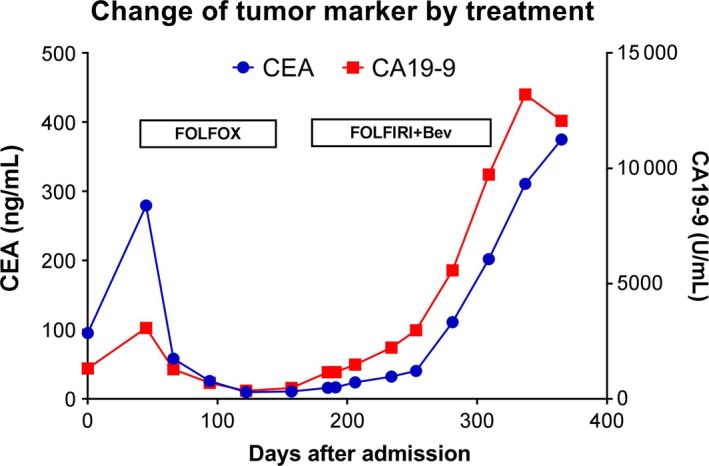
Changes of tumor markers in the patient during chemotherapy. While tumor markers carcinoembryonic antigen and cancer antigen 19‐9 decreased for months in response to FOLFOX treatment, it increased consistently in response to FOLFIRI + Bev

**FIGURE 6:**
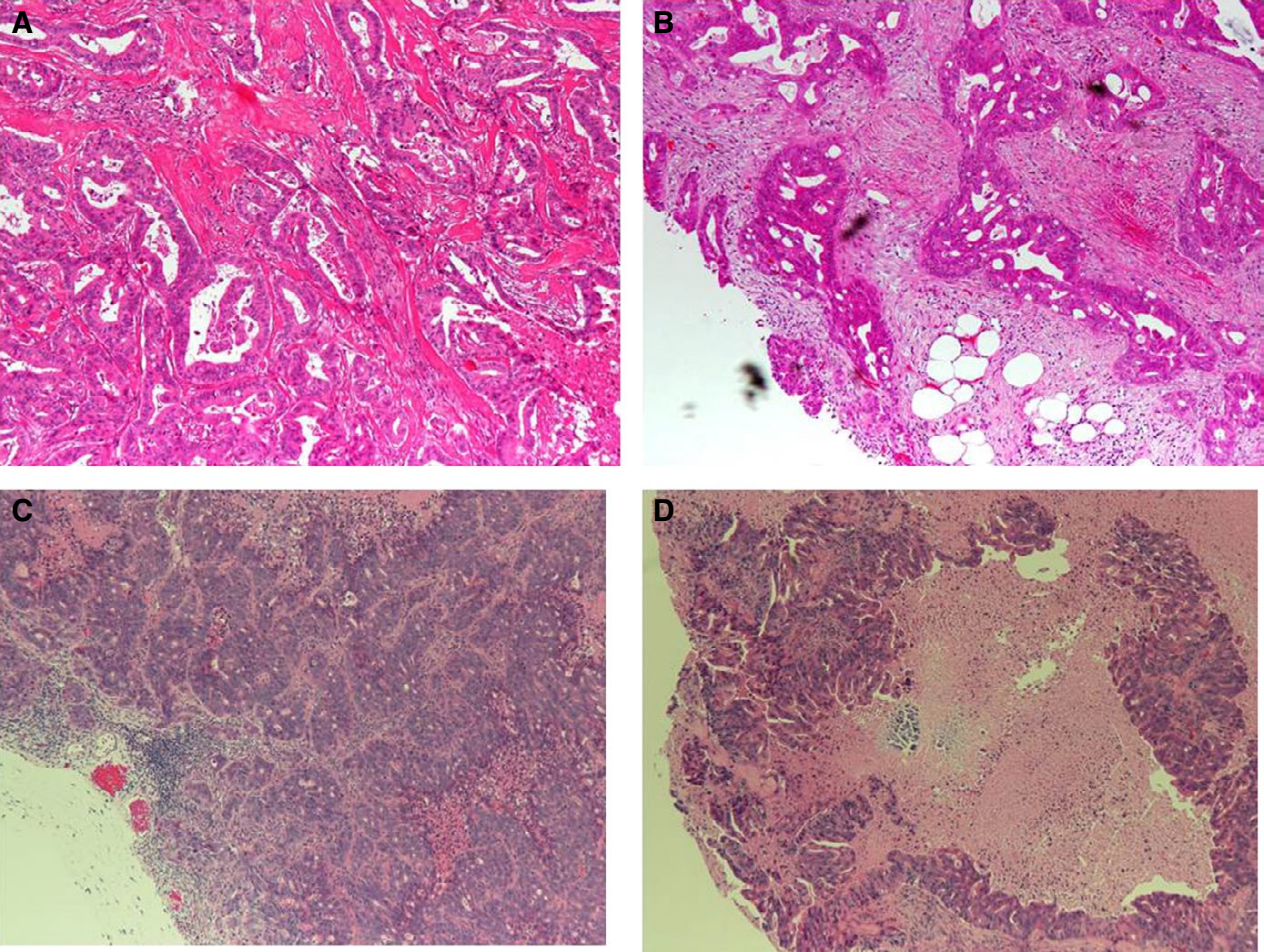
Hematoxylin–eosin staining of tumor samples. Shown is the staining of (A) the primary tumor, (B) metastatic peritoneal tumor, (C) patient‐derived xenograft (PDX) from the primary tumor, and (D) PDX from the metastatic peritoneal tumor. The pathological evaluation shows the presence of moderately differentiated tubular adenocarcinoma in the PDX, which is similar to that in the original tumor. Magnification (40×)

Five months after the initial treatment with FOLFOX, the patient was diagnosed with progressive disease (PD) by CT imaging. The imaging revealed an increase in tumor size of the liver, although the expression of the tumor markers continued to decrease (Figure 5). Treatment with FOLFOX was stopped, and she was started on FOLFIRI. However, the disease continued to progress, and the expression of tumor markers consistently increased, indicating that FOLFOX was more effective than FOLFIRI, which was consistent with our findings in the PDX models. Invasive growth of moderately differentiated adenocarcinoma was seen in the patient's primary tumor (Figure 6A), while moderately differentiated adenocarcinoma was seen in the patient's omental fat tissue (Figure 6B). In mice, the tumors derived from the patient's primary and peritoneal tumors had characteristics similar to the parent tumors as shown in Figure 6C,D. These findings indicate that the PDX tumors had inherited the morphological characteristics of the parent tumors from the patient.

### 3.5 Genomic assessment of the PDX model

Genetic analysis of the primary tumor, PDX (P4), and the PDX‐derived cell line has been summarized in Table 2 and Table S1. While microsatellite stability was consistent in these three samples, the mutations in them varied. First, all three samples showed *ALK, APC, CSF1R, EGFR, FGFR1, FGFR3, FLT3, KDR, HRAS, PDGFRA, RET,* and *TP53* mutations with high frequency, which were considered indispensable for tumor progression in this SBA model. Second, mutations in *NRAS, PTEN, KRAS, IDH2, STK11, ERBB$, SMRCB1, FBXW7,* and *NOTCH1* were detected only in the PDX. These genes with lower mutation frequencies bore 2‐8 mutation sites. Third, *KRAS* and *TP53* mutations were detected in the PDX and the cell line, but not in the primary tumor, which suggests that there might have been an increase of mutations in the PDX, which were not associated with proliferation. The genetic status of the PDX‐derived cell line was closer to the primary tumor than the PDX itself.

**TABLE 2:** Comparison of genetic status in the primary tumor, PDX tumor, and the SBA cell line

Genetic status	Primary tumor	PDX	Cell line
Microsatellite status	MSS	MSS	MSS
Mutation frequency in *CSF1R*, *FGFR3, FLT3, HRAS, RET, PDGFRA*	100%	100%	100%
Mutation frequency in *APC*	48%	100%	100%
Mutation frequency in *TP53*	46.2%	100%	100%
Mutation frequency in *ALK*	52.8%	69%	61.5%
Mutation frequency in *EGFR*	51.1%	51%	53.9%
Mutation frequency in *KDR*	46.7%	46.2%	47%
Mutation frequency in *KDR*	50.8%	50.5%	48.8%
Mutation frequency in *KDR*	60.5%	61.6%	60%
Mutation frequency in *KRAS*	(−)	100%	100%
Mutation frequency in TP53	(−)	15%	25%
Mutation in *ERBB4, FBXW7, KRAS IDH2, NOTCH1, PTEN, SMARCB1, STK11*	(−)	(+)	(−)

Note

Percentage indicates the mutation rate in indicated gene.

Abbreviations: PDX, patient‐derived xenograft; MSS, microsatellite stable.

## 4 DISCUSSION

To the best of our knowledge, we have, for the first time, demonstrated that the combination of OHP with 5‐FU is effective for the treatment of SBA in a preclinical PDX model.

SBA is a rare malignancy with a poor prognosis,,^4^, ^5^ and as a result of its rarity, there are limited randomized clinical trials involving SBA patients. Small retrospective and prospective phase II studies have demonstrated the effectiveness of the combination therapy with OHP and 5‐FU in these patients.^6^, ^7^, ^8^, ^9^, ^10^ However, these trials were not based on preclinical studies since there is a lack of appropriate preclinical and basic models for SBA. In this study, we have, for the first time, demonstrated the usefulness of this combination in an experimental model. Moreover, drug sensitivity studies demonstrated that OHP had a better IC_50_ value in PDX‐derived cells compared to the colon cancer cell line HT29, while irinotecan and 5‐FU had comparable IC_50_ values in the two cell types.

The genetic status of the PDX was different from those of the patient tumors as well as the PDX‐derived cell line, suggesting that the additional genetic mutations in the PDX might not be necessary for cell proliferation during the passages. *APC and TP53* mutations were consistent with previous reports on the genetic analysis of SBA.^23^, ^24^ Similarities in the genetic status of the patient tumor, and the PDX‐derived cell line suggests that the latter can be a good experimental tool for SBA research.

PDXs are preclinical models that can predict the drug sensitivities in the patients.^12^, ^13^, ^14^ Such models may, therefore, be required to overcome the problems associated with the rare nature of tumors such as SBA.^15^, ^16^ However, reports on PDX models of rare tumors are limited, perhaps because the cost–benefit ratio is unfavorable compared to that for common malignancies such as breast, lung, and colorectal cancers.

This study has several limitations. First, the PDX model was derived from a single patient and is not representative of all patients with SBA. Heterogeneity in SBAs is, therefore, not represented in this study. Further studies using a collection of PDXs are required to validate our findings. Second, this PDX model used immune‐deficient mice (nude mice), and the promising immune check‐point inhibitors were not available for this model.

The establishment of PDX models and derivative cell lines is potentially beneficial to the study of rare tumors. We expect this study to contribute to the project that collects PDX models of rare tumors, including SBAs.

In conclusion, we have established a PDX model from a primary SBA in a patient, and a cell line from the PDX. Assessment of drug sensitivity both in the PDX model and in vitro indicated that the combination of OHP and 5‐FU is an effective standard therapy for patients with advanced SBA. Such preclinical models are, therefore, useful in understanding the biology of such rare tumors.

## CONFLICT OF INTERESTS

The authors declare no conflicts of interest.

## AUTHOR CONTRIBUTIONS

TY conceived the study; TY performed experiments; TY, SK, and NT analyzed and interpreted data; TY wrote the manuscript; all authors reviewed the manuscript.

## Supporting information

Table S1

## Data Availability

The data that support the findings of this study are available from the corresponding author upon reasonable request.
