# Supplementary material for: A patient‐derived xenograft and a cell line derived from it form a useful preclinical model for small bowel adenocarcinoma
Source: Cancer Med. 2020 Mar 13;9(10):3337–43. doi: 10.1002/cam4.2986 (PMC7221307; doi:10.1002/cam4.2986)
Supplement: Supplementary file 1 — Table S1 [file CAM4-9-3337-s001.docx]

**TABLE S1**. Genetic status of PDX model

| **Sample** | **Chromosome** | **Position** | **Reference** | **Variant** | **Allele Call** | **Frequency (%)** | **Gene ID** |
| --- | --- | --- | --- | --- | --- | --- | --- |
| PDX | chr1 | 115258695 | T | C | Heterozygous | 27.6 | NRAS |
| PDX | chr1 | 115258722 | T | C | Heterozygous | 27.6 | NRAS |
| PDX | chr1 | 115258727 | GT | AG | Heterozygous | 27.2 | NRAS |
| Primary | chr10 | 43613843 | G | T | Homozygous | 100 | RET |
| PDX | chr10 | 43613843 | G | T | Homozygous | 100 | RET |
| SBA | chr10 | 43613843 | G | T | Homozygous | 100 | RET |
| Primary | chr10 | 43615633 | C | G | Heterozygous | 47.2 | RET |
| PDX | chr10 | 43615633 | C | G | Heterozygous | 51 | RET |
| SBA | chr10 | 43615633 | C | G | Heterozygous | 5.4 | RET |
| PDX | chr10 | 89692828 | T | C | Heterozygous | 8.8 | PTEN |
| PDX | chr10 | 89692906 | A | G | Heterozygous | 8.5 | PTEN |
| PDX | chr10 | 89720719 | A | G | Heterozygous | 31.8 | PTEN |
| PDX | chr10 | 89720734 | A | T | Heterozygous | 31.6 | PTEN |
| PDX | chr10 | 89720743 | A | G | Heterozygous | 31.6 | PTEN |
| Primary | chr11 | 534242 | A | G | Homozygous | 100 | HRAS |
| PDX | chr11 | 534242 | A | G | Homozygous | 100 | HRAS |
| SBA | chr11 | 534242 | A | G | Homozygous | 100 | HRAS |
| PDX | chr12 | 25380332 | C | T | Heterozygous | 29.1 | KRAS |
| PDX | chr12 | 25380344 | A | G | Heterozygous | 28.1 | KRAS |
| PDX | chr12 | 25398284 | C | T | Homozygous | 100 | KRAS |
| SBA | chr12 | 25398284 | C | T | Homozygous | 100 | KRAS |
| Primary | chr13 | 28610183 | A | G | Homozygous | 100 | FLT3 |
| PDX | chr13 | 28610183 | A | G | Homozygous | 100 | FLT3 |
| SBA | chr13 | 28610183 | A | G | Homozygous | 100 | FLT3 |
| PDX | chr15 | 90631873 | T | A | Heterozygous | 22 | IDH2 |
| PDX | chr15 | 90631879 | T | G | Heterozygous | 22.1 | IDH2 |
| PDX | chr15 | 90631900 | G | T | Heterozygous | 22.2 | IDH2 |
| PDX | chr15 | 90631906 | C | T | Heterozygous | 22 | IDH2 |
| PDX | chr15 | 90631908 | G | T | Heterozygous | 22 | IDH2 |
| PDX | chr15 | 90631915 | A | G | Heterozygous | 22.7 | IDH2 |
| PDX | chr15 | 90631918 | CCCCCCC | TCCCCCA | Heterozygous | 21.9 | IDH2 |
| PDX | chr15 | 90631939 | A | C | Heterozygous | 22.3 | IDH2 |
| PDX | chr17 | 7578556 | T | A | Heterozygous | 15 | TP53 |
| SBA | chr17 | 7578556 | T | A | Heterozygous | 25 | TP53 |
| Primary | chr17 | 7579472 | G | C | Heterozygous | 46.2 | TP53 |
| PDX | chr17 | 7579472 | G | C | Homozygous | 100 | TP53 |
| SBA | chr17 | 7579472 | G | C | Homozygous | 100 | TP53 |
| PDX | chr19 | 1206984 | G | C | Heterozygous | 34.3 | STK11 |
| PDX | chr19 | 1207014 | C | A | Heterozygous | 34.3 | STK11 |
| PDX | chr19 | 1207035 | G | A | Heterozygous | 34.6 | STK11 |
| PDX | chr19 | 1207038 | G | C | Heterozygous | 34.6 | STK11 |
| PDX | chr19 | 1207077 | G | C | Heterozygous | 34.2 | STK11 |
| PDX | chr19 | 1207083 | A | G | Heterozygous | 34.1 | STK11 |
| PDX | chr19 | 1207089 | T | G | Heterozygous | 33.9 | STK11 |
| Primary | chr2 | 29443617 | C | G | Heterozygous | 52.8 | ALK |
| PDX | chr2 | 29443617 | C | G | Heterozygous | 69 | ALK |
| SBA | chr2 | 29443617 | C | G | Heterozygous | 61.5 | ALK |
| PDX | chr2 | 212288931 | A | G | Heterozygous | 20 | ERBB4 |
| PDX | chr2 | 212288935 | T | C | Heterozygous | 18.6 | ERBB4 |
| PDX | chr2 | 212288947 | T | C | Heterozygous | 19.3 | ERBB4 |
| PDX | chr2 | 212288949 | A | G | Heterozygous | 19.2 | ERBB4 |
| PDX | chr2 | 212288956 | A | G | Heterozygous | 19.1 | ERBB4 |
| PDX | chr2 | 212288968 | C | G | Heterozygous | 19.2 | ERBB4 |
| PDX | chr22 | 24133957 | C | G | Heterozygous | 37 | SMARCB1 |
| PDX | chr22 | 24134003 | A | C | Heterozygous | 38.1 | SMARCB1 |
| PDX | chr22 | 24134026 | G | A | Heterozygous | 37 | SMARCB1 |
| PDX | chr22 | 24134041 | T | G | Heterozygous | 36.9 | SMARCB1 |
| Primary | chr4 | 1803614 | G | A | Heterozygous | 52.6 | FGFR3 |
| PDX | chr4 | 1803614 | G | A | Heterozygous | 52.4 | FGFR3 |
| SBA | chr4 | 1803614 | G | A | Heterozygous | 54.4 | FGFR3 |
| Primary | chr4 | 1807894 | G | A | Homozygous | 100 | FGFR3 |
| PDX | chr4 | 1807894 | G | A | Homozygous | 100 | FGFR3 |
| SBA | chr4 | 1807894 | G | A | Homozygous | 100 | FGFR3 |
| Primary | chr4 | 55141055 | A | G | Homozygous | 100 | PDGFRA |
| PDX | chr4 | 55141055 | A | G | Homozygous | 100 | PDGFRA |
| SBA | chr4 | 55141055 | A | G | Homozygous | 100 | PDGFRA |
| Primary | chr4 | 55962546 | - | G | Heterozygous | 60.5 | KDR |
| PDX | chr4 | 55962546 | - | G | Heterozygous | 61.6 | KDR |
| SBA | chr4 | 55962546 | - | G | Heterozygous | 60 | KDR |
| Primary | chr4 | 55972974 | T | A | Heterozygous | 50.8 | KDR |
| PDX | chr4 | 55972974 | T | A | Heterozygous | 50.5 | KDR |
| SBA | chr4 | 55972974 | T | A | Heterozygous | 48.8 | KDR |
| Primary | chr4 | 55980239 | C | T | Heterozygous | 46.7 | KDR |
| PDX | chr4 | 55980239 | C | T | Heterozygous | 46.2 | KDR |
| SBA | chr4 | 55980239 | C | T | Heterozygous | 47 | KDR |
| PDX | chr4 | 153250881 | T | G | Heterozygous | 12.4 | FBXW7 |
| PDX | chr4 | 153250887 | A | G | Heterozygous | 12.3 | FBXW7 |
| PDX | chr4 | 153250901 | A | G | Heterozygous | 12.3 | FBXW7 |
| PDX | chr4 | 153250917 | A | G | Heterozygous | 12.2 | FBXW7 |
| PDX | chr4 | 153250926 | T | C | Heterozygous | 10.8 | FBXW7 |
| Primary | chr5 | 112175770 | G | A | Heterozygous | 48 | APC |
| PDX | chr5 | 112175770 | G | A | Homozygous | 100 | APC |
| SBA | chr5 | 112175770 | G | A | Homozygous | 100 | APC |
| Primary | chr5 | 149433596 | TG | GA | Homozygous | 100 | CSF1R |
| PDX | chr5 | 149433596 | TG | GA | Homozygous | 100 | CSF1R |
| SBA | chr5 | 149433596 | TG | GA | Homozygous | 100 | CSF1R |
| Primary | chr7 | 55249063 | G | A | Heterozygous | 51.1 | EGFR |
| PDX | chr7 | 55249063 | G | A | Heterozygous | 51 | EGFR |
| SBA | chr7 | 55249063 | G | A | Heterozygous | 53.9 | EGFR |
| Primary | chr8 | 38285911 | GA | TC | Heterozygous | 50.3 | FGFR1 |
| PDX | chr8 | 38285911 | GA | TC | Heterozygous | 35.2 | FGFR1 |
| SBA | chr8 | 38285911 | GA | TC | Heterozygous | 42.4 | FGFR1 |
| PDX | chr9 | 139399350 | C | T | Heterozygous | 6.3 | NOTCH1 |
| PDX | chr9 | 139399367 | G | A | Heterozygous | 6.2 | NOTCH1 |
| PDX | chr9 | 139399380 | C | T | Heterozygous | 6 | NOTCH1 |
| PDX | chr9 | 139399385 | G | C | Heterozygous | 6.1 | NOTCH1 |
| PDX | chr9 | 139399388 | C | T | Heterozygous | 6.5 | NOTCH1 |

PDX, patient derived xenograft, SBA, small bowel adenocarcinoma cell
